# Supplementary material for: Significant Variations in Double-Stranded RNA Levels in Cultured Skin Cells
Source: Cells. 2024 Jan 25;13(3):226. doi: 10.3390/cells13030226 (PMC10854852; doi:10.3390/cells13030226)
Supplement: Supplementary file 1 [file cells-13-00226-s001.zip › cells-2768577-supplementary.pdf]

Supplementary Material

Supp. Table 1	Primers
Supp. Table 2	Read counts
Supp. Figure 1	Biotypes
Supp. Figure 2	Compiled protein blots for Figure 6B
	Original blots
Supp. Figure 3	Mitochondrial dsRNA

Supp. Table 1 Primers for dsRNA sensors

Gene name	dsRNA BINDING PROTEIN	Primer sequence, 5' to 3'	Annealing temp.	Product length
Eukaryotic translation initiation factor 2A, 65kDa	PKR	Sense= ACTGTGGACAAGAGGTTTGG<br>Antisense=GCTCCGCCTTCTCGTTATTAT	58°C	148bp
DDX58	RIG	Sense= TGTGGGCAATGTCATCAAAA [59]<br>Antisense=GAAGCACTTGCTACCTCTTGC [59,60]	58°C	67bp
IFIH1 Interferon induced with helicase C domain 1	MDA-5	Sense= GGCACCATGGGAAGTGATT [60]<br>Antisense=ATTTGGTAAGGCCTGAGCTG[60]	58°C	86bp
Adenosine deaminase	ADAR	Sense= GCTTGGGAACAGGGAATCG [61]<br>Antisense=CTGTAGAGAAACCTGATGAAGCC [3]	58°C	118bp
ACTIN	ACTIN	Sense= AGAGCTACGAGCTGCCTGAC<br>Antisense= AGCACTGTGTTGGCGTACAG	58°C	184bp

Supp. Table 2 Statistics of RNA sequencing data

Sequencing types	Cell types	Rep	Total Reads	Trimmed Reads	% Human reads	%Zebrafish reads
Total RNA INPUT sequencing	A375	1	43,952,677	43,823,919	46.84%	-
		2	42,306,724	42,174,665	49.30%	-
		3	39,312,508	39,184,473	56.17%	-
	C8161	1	19,729,146	19,652,500	65.45%	-
		2	26,015,355	25,919,440	63.31%	-
		3	25,397,350	25,292,915	60.59%	-
	Fibroblast	1	26,588,838	26,486,894	67.56%	-
		2	27,397,425	27,301,503	70.47%	-
		3	24,286,285	24,202,846	68.16%	-
dsRNA sequencing	A375	1	27,862,692	27,775,409	54.98%	8.9915
		2	24,285,054	23,947,964	18.34%	26.5553
		3	21,525,075	21,439,872	33.53%	39.0859
	C8161	1	33,981,195	33,912,385	3.94%	93.8973
		2	30,902,661	30,837,649	3.97%	94.2203
		3	22,313,531	22,269,720	5.09%	88.2593
	Fibroblast	1	56,528,406	28,205,788	0.00%	99.9445
		2	35,540,230	35,471,576	0.38%	99.7678
		3	39,446,736	39,377,513	0.44%	99.4715

Supp. Figure 1 **Biotypes**

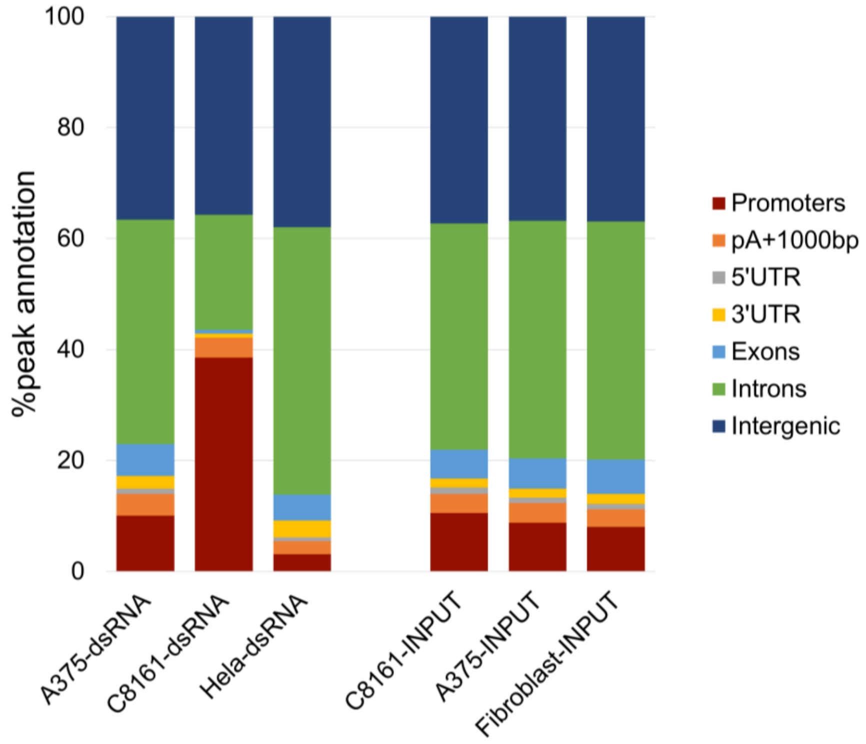

Supplementary Figure 1. Expression analysis using a peak annotation pipeline. In brief, a base-line expression was established using all aligned reads. Peaks were called when read numbers exceeded five times the back and categorized into promoter (-1000 to TSS), 5' UTR, 3'UTR, Exon, Intron, polyA signal +1000 and intergenic regions [22]. Percentages of the reads in the different categories are given. Remarkably, the proportion of biotypes remained rather stable between cell types and also between dsRNA immune enriched (-dsRNA) and input samples. The only notable example is C8161-dsRNA which shows a high percentage of promoter associated reads.

Supp. Figure 2 **Compiled protein blots for Figure 6B**

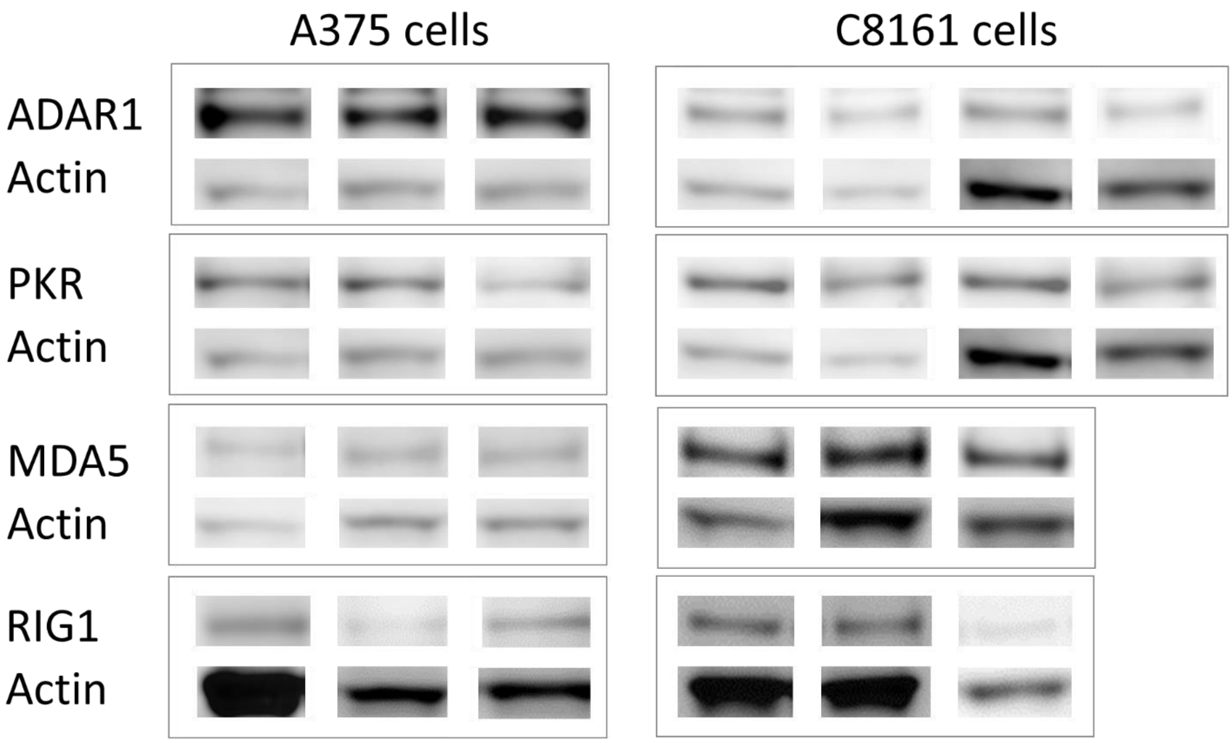

Supplementary Figure 2. Western blot bands of dsRNA sensors (ADAR1, PKR, MDA5 and RIG1) including actin as internal standard. Membranes were cut to assess the same blot for proteins with different molecular weight. Blot 1, ADAR1 110 kD; PKR 68 kD; actin 42 kD. Blot 2, MDA5 117 kD and actin 42 kD. Blot 3, RIG1 107 kD actin 42 kD. The band were quantified using image FIJI software and results are summarized in Figure 6B.

Original blots

A375 cells, MDA5

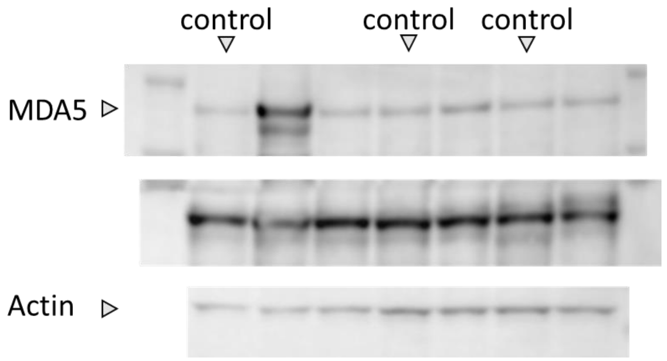

C8161 cells, MDA5

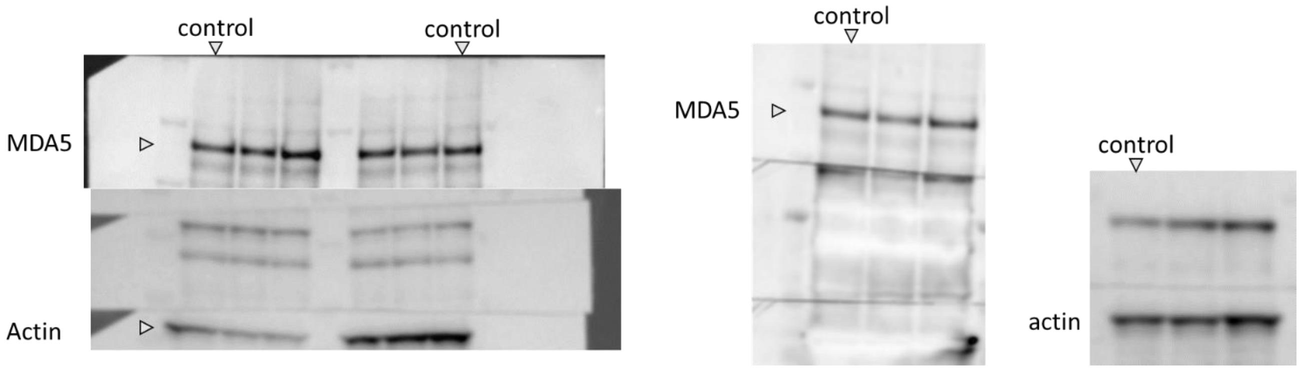

A375 cells, ADAR1 and PKR

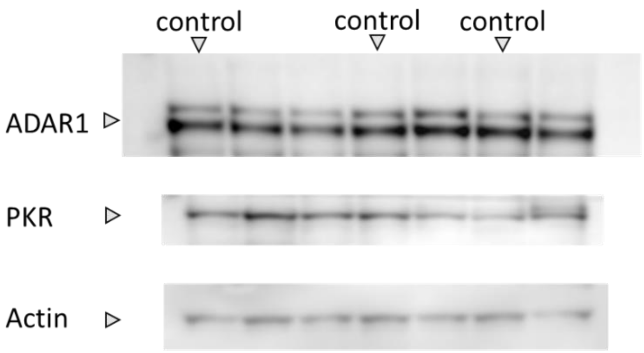

C8161 cells, ADAR1 and PKR

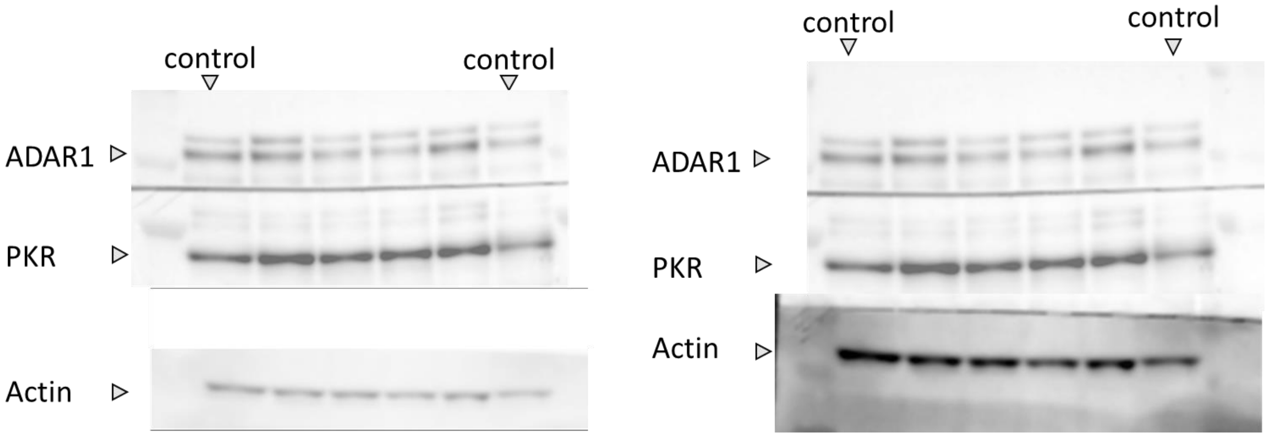

A375 cells, RIG1

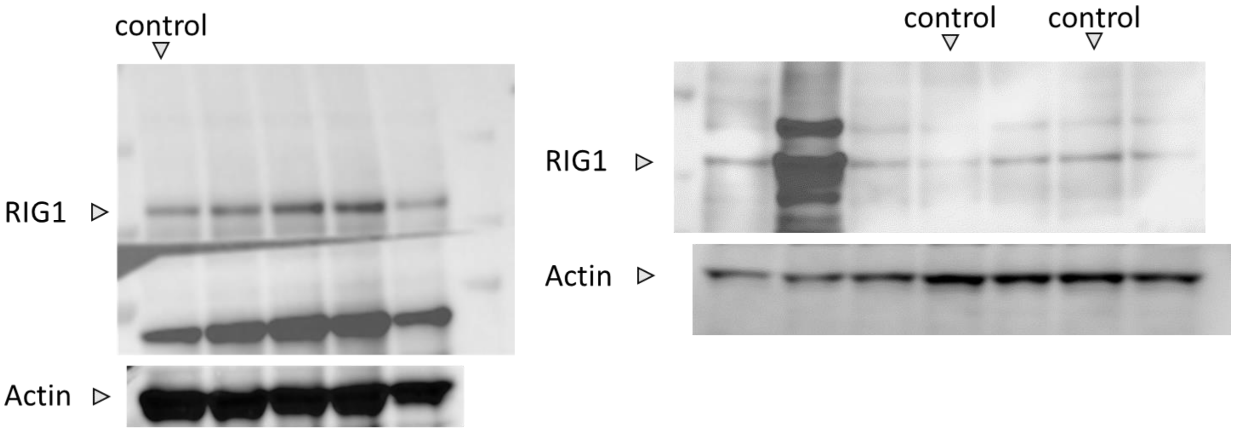

C8161 cells, RIG1

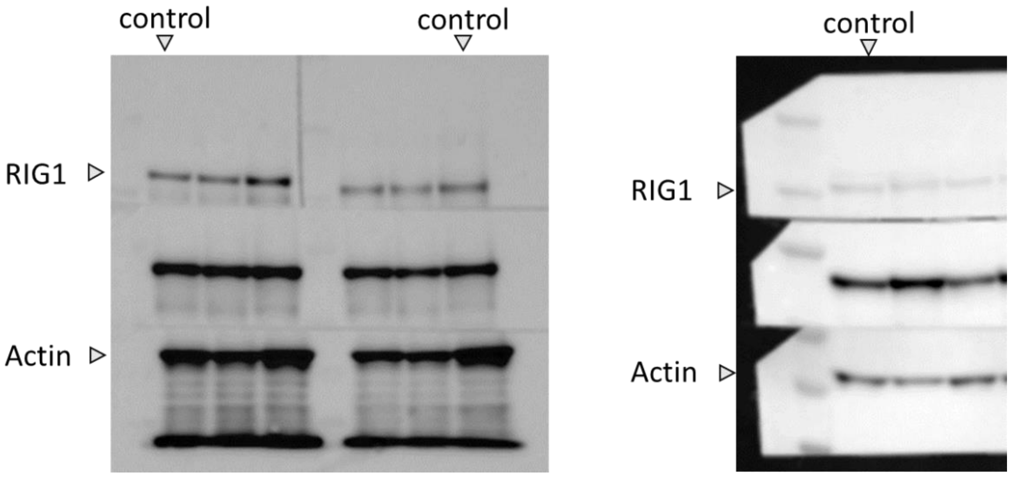

Original blots for Supplementary Figure 2. The bands representing the dsRNA sensors (ADAR1, PKR, MDA5 and RIG1) and actin in the lanes labeled 'control' were quantified for Figure 6B and cropped and compiled into Supplementary Figure 2.

Supp. Figure 3 **Mitochondrial dsRNA**

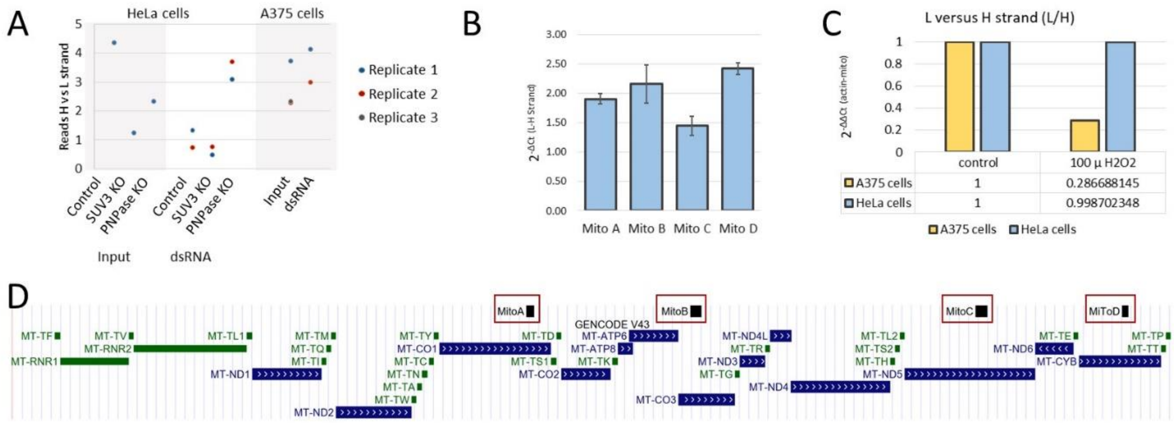

Supplementary Figure 3. Assessment of dsRNA in mitochondria. (**A**) Comparison of RNAseq data from Hela cells [3] and A375 cells, dsRNA enriched samples and input control. The quotient of total read numbers heavy versus light strand is presented. The number of replicates varied between experiments. In Hela cells, two enzymes that degrade the light strand (SUV3 and PNPase) were knocked down as indicated. (**B**) RT-qPCR based comparison of heavy strand versus light strand levels. All primers revealed close to a twofold excess of heavy versus light strand. Two biological, each 3 technical replicates. Primer pair D was used for further analysis in figure E. (**C**) Consequences of H_2O_2 stress on the levels of mitochondrial light and heavy strand RNA. Exposure to 100 µM H_2O_2 had a clear effect on the proportion of light versus heavy strand in A375 cells (reduction to about 28%), in contrast the stress had little influence on the proportion of strands in HeLa cells (the quotient in control cells is set as 1, compared to RNA levels after treatment). The difference between replicates was less than 0.22 cycles. (**D**) Position of the assessed primer pairs in the mitochondrial genome. The sequence of the primers used is given in the table below.

For RT-PCR of mitochondria RNA several steps were necessary to ensure strand specific amplification mitochondrial RNA. First, the RNA was DNAse treated. Second, strand-specific primers with an adaptor sequence were used for RT. Third, the RT reaction was digested with Exonuclease I before dilution and qPCR and fourth, the qPCR was run with the adaptors primers (Ad1 and Ad2) [62]. In all reactions an RNA control was included and only reactions with a difference of at least 5 cycles between cDNA and RNA were included. Adaptor sequences are in italic in the table below.

Name	Bases	Sequence	
Ad1-MTAfor	41	*GGC CGT CAT GGT GGC GAA TAA* CCG GAT AGG CCG AGA AAG TG	
Ad2-MTArev	40	*CGT GGC GAA CGA CAG TAA TA*T CAG GCT ACA CCC TAG ACC A	
Ad1-MTBfor	41	*GGC CGT CAT GGT GGC GAA TAA* GGC TGG AGT GGT AAA AGG CT	
Ad2-MTBrev	40	*CGT GGC GAA CGA CAG TAA TA*A CCA ATG ATG GCG CGA TGT A	
Ad1-MTCfor	41	*GGC CGT CAT GGT GGC GAA TAA* TCC TGC TAA TGC TAG GCT GC	
Ad2-MTCrev	40	*CGT GGC GAA CGA CAG TAA TA*A CAT CTG TAC CCA CGC CTT C	
Ad1-MTDfor	41	*GGC CGT CAT GGT GGC GAA TAA* CGA GGG CGT CTT TGA TTG TG	
Ad2-MTDrev	40	*CGT GGC GAA CGA CAG TA*A TAT CTT GCA CGA AAC GGG ATC A	
Ad1	21	*GGC CGT CAT GGT GGC GAA TAA*	Tm 60.3
Ad2	20	*CGT GGC GAA CGA CAG TAA TA*	Tm 54.8
